# Analysis of Parasitic Protozoa at the Single-cell Level using Microfluidic Impedance Cytometry

**DOI:** 10.1038/s41598-017-02715-y

**Published:** 2017-06-01

**Authors:** J. S. McGrath, C. Honrado, D. Spencer, B. Horton, H. L. Bridle, H. Morgan

**Affiliations:** 1 Institute of Biological Chemistry, Biophysics and Bioengineering, School of Engineering and Physical Sciences, Heriot-Watt University, Edinburgh, EH14 4AS United Kingdom; 2 Faculty of Physical Sciences and Engineering and Institute for Life Sciences, University of Southampton, Southampton, SO17 1BJ United Kingdom; 3 Moredun Scientific, Pentlands Science Park, Bush Loan, Penicuik, Midlothian, EH26 0PZ United Kingdom

## Abstract

At present, there are few technologies which enable the detection, identification and viability analysis of protozoan pathogens including *Cryptosporidium* and/or *Giardia* at the single (oo)cyst level. We report the use of Microfluidic Impedance Cytometry (MIC) to characterise the AC electrical (impedance) properties of single parasites and demonstrate rapid discrimination based on viability and species. Specifically, MIC was used to identify live and inactive *C. parvum* oocysts with over 90% certainty, whilst also detecting damaged and/or excysted oocysts. Furthermore, discrimination of *Cryptosporidium parvum*, *Cryptosporidium muris* and *Giardia lamblia*, with over 92% certainty was achieved. Enumeration and identification of (oo)cysts can be achieved in a few minutes, which offers a reduction in identification time and labour demands when compared to existing detection methods.

## Introduction

The protozoan pathogens *Cryptosporidium* and *Giardia* are responsible for a large, global disease burden which affects the health of both humans and livestock and, in turn, has a significant knock-on economic impact^1–3^. Globally, the human disease burden for *Cryptosporidium* is estimated at 250–500 million cases per year^1^. A waterborne outbreak of cryptyosporidiosis in Milwaukee in 1993 infected 400,000 people with a cost of $96 million^3, 4^ and outbreaks have occurred more recently in Australia^5^, the UK^6^ and Sweden^7^. In the UK, an outbreak of *Cryptosporidium* in Lancashire, beginning August 2015, is estimated to have affected 300,000 households over a six-week period with a cost of approx. £15 m^6^. With the global population continuing to rise, the intensification of farming and the growing demand for potable drinking-water will ﻿li﻿kely lead to an increase in the incidence of disease in humans and animals^1, 4^. Food- and water-borne pathogens are therefore of growing concern to governments and industry worldwide.

*Cryptosporidium* oocysts are produced in high numbers, highly infective, persistent in water, resistant to chlorination, deformable and able to evade modern filtration systems in low numbers^2, 4, 8–12^. With fewer than 10 oocysts capable of initiating a significant human infection^13, 14^, waterborne monitoring for the presence of this pathogen is therefore essential^15, 16^. The standard method of isolation and detection for *Cryptosporidium* oocysts present in treated water supplies, EPA 1623.1^17^, typically involves the processing of 50–100 L of water (*e.g*., on site) or 1000 L (*e.g*., at the treatment works) to a 50 µL volume, before highly-skilled microscopists confirm detection using specialised techniques^18^. This process also simultaneously recovers cysts of the *Giardia* genus – protozoan parasites that may also cause gastroenteritis if ingested by humans^17^.

The ability to rapidly enumerate and discriminate *Cryptosporidium* oocysts (*e.g*., post-recovery *via* EPA 1623.1) based on viability status and/or species in an automated process would reduce detection time, reduce the level of human intervention required, aid in better assessing the risk posed to human health and contribute to the saving of resources^19^. To discern between live and temperature-inactivated oocysts, the intrinsic electrical properties of single *C. parvum* have been measured using AC electrokinetic techniques such as dielectrophoresis^20, 21^ and/or electrorotation^22, 23^. Measurements were either performed in batch^20, 22, 23^, required long processing times^21^ or required microscopy to assess the oocyst response to an applied field^20–23^. AC electrokinetic techniques have been utilised for sorting at the genus level, *e.g. Cryptosporidium* and *Giardia*. Unni *et al*.^24^ demonstrated dielectrophoretic separation of *Cryptosporidium muris* and *Giardia lamblia* on-chip in batch mode, but did not report a limit of detection. However, such approaches have not, to our knowledge, been used for the discrimination of alternate species of *Cryptosporidium* and it has been suggested that the separation of *C. parvum* and *C. muris* might not be possible, as both show only minor difference in dielectric properties^24^.

The only use of an electrical impedance analysis system to characterise the electrical properties of viable and non-viable *C. parvum* oocysts was demonstrated by Houssin *et al*.^25^. Results showed a 15% difference in impedance magnitude with a low conductivity buffer containing either viable or non-viable *C. parvum*, with a reported detection limit <10 oocysts µL^−1^. Measurements were made in bulk, where aliquots containing oocysts, either viable or non-viable, were loaded into sample wells, with the impedance measured and compared to the control (previously measured values of the suspending fluid alone). However, oocysts in any real sample, *e.g*., those recovered using EPA 1623.1, could be of mixed viability; therefore, it is a necessity that any impedance system used for detection can discern the viability status of single oocysts. To our knowledge, there are no reports of the impedance analysis of different *Cryptosporidium* species, or the simultaneous impedance analysis of *Cryptosporidium* and *Giardia*.

Microfluidic Impedance Cytometry (MIC) is a microfluidic system that uses microelectrodes to measure the impedance of single particles^26, 27^. Suspended particles flow through a microchannel at high speed (flow rates up to ~80 µL min^−1^ can be used) and as they pass a detection region, the measured current gives a signal which is proportional to particle impedance. This method is label-free, requires minimal sample processing and measures up to 1000 particles per second^28^. In comparison to other continuous microfluidic separation/discrimination technologies^29, 30^, MIC offers rapid sample processing and high throughput. MIC has been used for discriminating various cell types including stem cells^31^, leukocytes^32, 33^ and tumour cells in whole blood^34^. However, the applicability of MIC to the detection of parasitic protozoa has not yet been widely explored and previous work has focussed on analysis of infected cells rather than direct protozoan analysis^35, 36^.

In this paper, we describe the use of MIC to detect single protozoan (oo)cysts within a continuous flow system. We characterise the biophysical properties of live and temperature-inactivated, human-pathogenic parasites and assess sample composition by analysing the electrical data. We also detect single *C. parvum* and *C. muris* oocysts, as well as *G. lamblia* cysts. Detection and viability/species analysis of oocysts in continuous flow using MIC offers a significant advance over other approaches, both in terms of gathering information at the single (oo)cyst level and the speed of data collection.

## Materials and Methods

### System Overview

The microfluidic impedance chips were fabricated as described previously^29, 34, 37^ using photolithography and full wafer thermal bonding. In short, platinum electrodes (30 µm width and 200 nm thickness) were patterned on a glass wafer. Microchannels, with cross-sectional dimensions 200 µm × 30 µm leading into a detection region of 40 µm × 30 µm, were defined in SU8 photoresist before thermal bonding of two wafers. After dicing, inlet and outlet channels were etched using a CO_2_ laser (Mini 18, Epilog Laser). The chips were held in a 3D-printed holder, which housed fluidic attachments and electrical connections. The holder was mounted on an *x*-*y*-*z* stage with optics to image the detection region. A syringe pump (Fusion 400, Chemyx) was used to introduce the sample from a syringe at a flow rate of 40 µL min^−1^.

**Figure 1:**
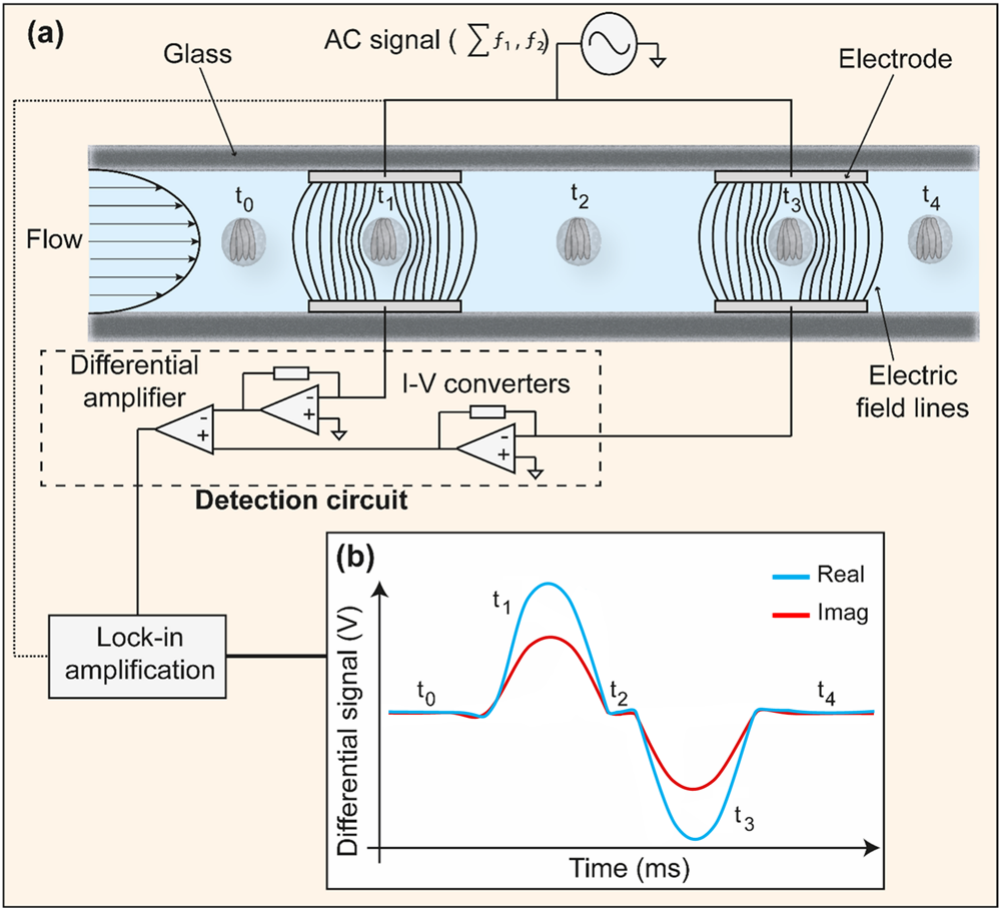
Schematic showing the microfluidic impedance cytometer. **(a)** The detection region consists of pairs of parallel facing electrodes which are 30 μm wide and separated by 50 μm. The electrodes are fabricated within a microfluidic channel which is 40 μm wide × 30 μm high. Pathogens suspended in PBS (*C. parvum* illustrated) are driven through the channel by pressure-driven flow. Sinusoidal voltages at two discrete frequencies (ƒ_1_, ƒ_2_) are applied to the top electrodes and the difference in current flowing through the bottom electrodes is measured by the custom detection circuit. The circuit consists of transimpedance amplifiers, which convert current (I) to voltage (V), and a differential amplifier. The response at each applied frequency is demodulated using lock-in amplification (HF2LI, Zurich Instruments). (**b**) The idealised differential signal when an oocyst passes through the centre of the detection region; the steps of the sequence are annotated (t_0_, t_1_, t_2_, t_3_ and t_4_).

Figure 1 shows a diagram of the impedance chip. Sinusoidal voltages at two simultaneous frequencies were applied to the top two electrodes using a digital impedance analyser (H2FLI, Zurich Instruments). The first frequency, termed the reference frequency, was applied at 18.3 MHz, while the second (probe) frequency was varied in the range 250 kHz to 50 MHz (Fig. 1). The voltage ranged from 1.5 Vpp to 10 Vpp depending on buffer conductivity and signal frequency. At frequencies above 20 MHz the signals are attenuated by the input and output filters of the lock-in amplifier, reaching −6dB at 50 MHz. Therefore, for frequencies ≥20 MHz, the applied voltage was increased incrementally to account for this attenuation. The average signal magnitude for the nearest 3 probe frequencies below 20 MHz was first calculated$\,({\bar{Z}}_{ < 20MHZ})$. Then, the gain was calculated by dividing this average value by the average magnitude signal for each frequency (e.g. $facto{r}_{50MHZ}=\,{\bar{Z}}_{ < 20MHZ}/{\bar{Z}}_{50MHZ}$ for 50 MHz). An example can be found in Supplementary Fig. S1. Increasing the voltage minimized signal loss without affecting data collection or analysis. At any one probe frequency, data was recorded for approximately 30 s, before the probe frequency was increased. The current flowing through both bottom electrodes was converted to voltage using transimpedance amplifiers. A differential amplifier was used to retrieve a single output signal. Lock-in amplification (HF2LI, Zurich Instruments) was used to separate the magnitude and phase at each frequency, from which impedance scatter plots were generated.

Between samples, the chips were flushed with 1 mL ethanol (70%) and 1 mL DI water using a syringe. Electrode surfaces were cleared of debris on a daily basis by introducing 1 M sodium hydroxide into the system at 50 µL min^−1^ for ~4 mins, before rinsing with 1 mL DI water.

### Sample Preparation

Four different stock samples were used in our analysis of untreated and heat-inactivated *C. parvum*. These samples, all obtained in suspensions containing PBS + 2% antibiotics + 0.01% Tween 20, were as follows:

- A batch of calf-sourced *C. parvum* (WC1) obtained from Waterborne Inc. (Iowa strain; US) was used within one month of propagation.
- A second batch of calf-sourced *C. parvum* (WC2) and a batch of mouse-sourced *C. parvum* (WM) from Waterborne Inc. (Iowa strain) were used within 1–2 months of oocyst propagation.
- Calf-sourced *C. parvum* (MC) obtained for Moredun Scientific (Moredun Strain; UK) were used when aged approximately 2–3 months.

From each stock, samples containing untreated or heat-inactivated *C. parvum* were independently prepared and the impedance measured separately. In each sample, oocysts were diluted to a final concentration of ~100 oocysts µL^−1^ of PBS. The effect of varying buffer conductivity was examined using different PBS concentrations, ranging from 0.76–7.10 S m^−1^. Each buffer also contained 0.1% Tween 20. Calibration polystyrene beads of 7 µm diameter (Microparticles GmbH) were added to each sample to a final concentration of 100 beads µL^−1^. Polystyrene beads have a known size and electrical properties^34^, and have a constant impedance across the frequency range used in these experiments. The measured impedance signal of each oo(cyst) was normalised with respect to the bead by dividing by the mean impedance of the polystyrene beads for each frequency, thus removing any non-linearities.

For impedance analysis of live *Cryptosporidium spp*. and *G*. *lamblia*, individual samples containing either *C*. *parvum*, *C*. *muris* or *G. lamblia* (obtained from Waterborne Inc.) were prepared in PBS + 0.1% Tween 20 (σ_m_ = 1.6 S m^−1^). Mixed samples were diluted to contain a total of ~100 (oo) cysts and 100 beads µL^−1^ of sample; mixed samples were prepared to include equal numbers of each pathogen. All samples were introduced to the system and measured as described previously.

### Oocyst Inactivation

In order to inactivate oocysts, samples prepared as described for untreated *C. parvum* in the previous section were subjected to heat-treatment at 70 °C for 5 mins in a heating block (Stuart Scientific, UK) before measurement. This treatment is reported in the literature as the minimum treatment required to induce loss of infectivity in the mouse model^20, 38^.

### Data Analysis

A custom script was written in Matlab (R2016a) for data processing and statistical analysis. In order to make reliable statistical inference regarding each population, samples containing each population were first measured separately. Using the comparison of untreated and heat-inactivated *C. parvum* as an example, the impedance signal was first normalised relative to the frequency-independent impedance response of the polystyrene beads. Then the *C. parvum* oocyst populations were gated from smaller debris and the larger reference beads using normalised impedance data gathered at the reference frequency (18.3 MHz). Upon gating, the normalised impedance response of gated pathogens at probe frequency were then be plotted and analysed.

By considering the spread and orientation of the data and assuming normal data distribution, it was possible to estimate the level of discrimination achieved across both impedance dimensions (real and imaginary, or opacity and size), at a given frequency, between any two populations. Firstly, the covariance matrix was calculated to identify the directions in which the 2-D data of a single population varied most – the largest and smallest eigenvectors of this matrix indicate the direction of the data spread and the magnitude of this spread was given by the eigenvalues of the matrix. Secondly, with respect to spread and orientation of each population, confidence ellipses containing all events within 1–3 standard deviations of the mean were plotted to identify the positions of equal probability deviation between two clusters, *i.e* the intersection of the equivalent confidence ellipses defines the boundary where a data point has equal probability of belonging to either population. A line of equal probability deviation was then fitted to the points of intersection and subsequently, the discrimination confidence was indicated – *i.e*., by counting the number of oocysts from each population that plotted either side of the line of equal probability deviation.

### Excystation Assay

The viability rates of the live *C. parvum* stock samples were estimated using an *in vitro* excystation assay developed at Moredun Research Institute (UK)^39^, to evaluate original stock sample quality and provide a reference for comparison of untreated and heat-inactivated *C. parvum*. Upon completion of the assay, oocysts were visualised using a differential interference contrast (DIC) microscope (BX50, Olympus) and the number of intact oocysts and empty shells were counted until 250 events had been recorded. For each sample, three counts were performed. The following formula was used to calculate excystation percentage: number of empty shells/(number of empty shells + number intact oocysts). The average sample viability rates for the original *C. parvum* stock samples were: 95% for WC1, 86% for WC2; 87% for WM; and 88% for MC. Please see Table S1 for more detailed excystation assay results.

### Optical Flow Cytometry

Conventional flow cytometric analysis of parasite suspensions was carried out at 35 µL min^−1^ using a BD Accuri C6 flow cytometer (BD Biosciences) with two lasers (488 nm and 640 nm). Data were exported as standard FCS files and the forward and side scattered light (FSC and SSC) signals were analysed using Matlab.

### Data availability

All data supporting this study are openly available from the University of Southampton repository at http://doi.org/10.5258/SOTON/D0047.

## Results and Discussion

### Dielectric properties

**Figure 2:**
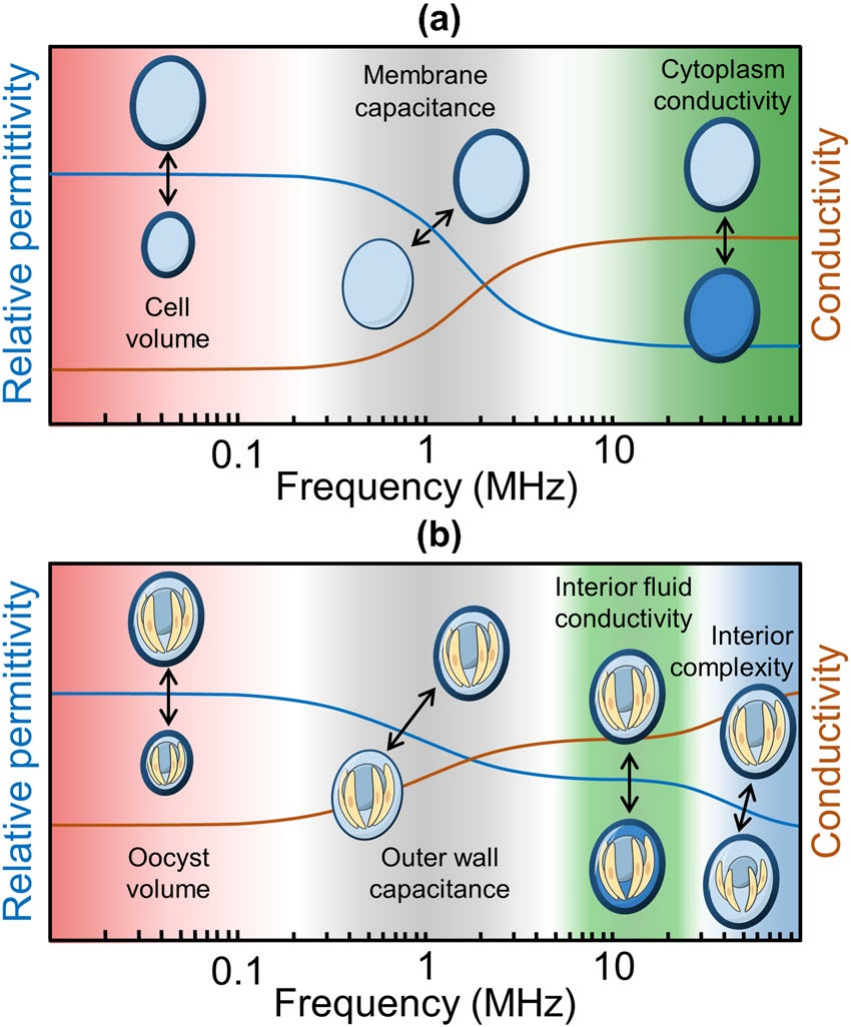
Illustration of the frequency-dependent dielectric response of a single-shelled particle or a *C. parvum* oocyst. (**a**) A simplified, single shell model is often used to approximate the dielectric properties of cells/particles. A cell/particle which is surrounded by a shell (membrane) experiences a single dielectric dispersion, within the frequency range of 1–10 MHz (called the β-relaxation). (**b**) For more complex biological structures, like the *C. parvum* oocyst, a simplified single-shell model cannot accurately calculate the dielectric properties of the oocyst components. For example, *C. parvum* oocysts are likely to experience multiple relaxations due to the polarisation of successive shells as the frequency increases. Note that the frequency window for these relaxations depends on the conductivity of the suspending medium.

The simplest electrical model that describes the properties of a cell is the so called single-shelled model as shown in Fig. 2(a). Generally, biological cells are insulating at low frequencies (kHz), due to the presence of the lipid cell membrane, but become increasingly conductive at higher frequencies (MHz) due to capacitive coupling across the membrane^25, 34, 40^. Therefore, for a viable cell, the impedance at low frequencies (<1 MHz) measures cell volume, while intermediate frequencies (0.5–10 MHz) measures membrane capacitance, which reflects membrane invagination and convolution. At higher frequencies, the electric field capacitively couples across the membrane and the impedance signal reflects the cytoplasm properties and any nucleus which may be present. Figure 2(b) illustrates the specific case for an oocyst which contains four nuclei-containing sporozoites and various organelles. By analogy with a typical cell, at low frequencies only the volume of the intact oocysts can be measured. In the MHz regime, oocyst impedance firstly becomes a function of the outer wall capacitance (typically at frequencies 1–10 MHz) before the conductivity of the interior strongly influences the response at high frequencies (>10 MHz)^27^. Consequently, in the intermediate frequency range viable oocysts of equivalent size may show differences in their impedance due to variations in the composition and/or structure of the outer wall and the interior. As the signal frequency increases further, *i.e*., >30 MHz, the impedance is further influenced by the sporozoites and nuclei^27^.

**Figure 3:**
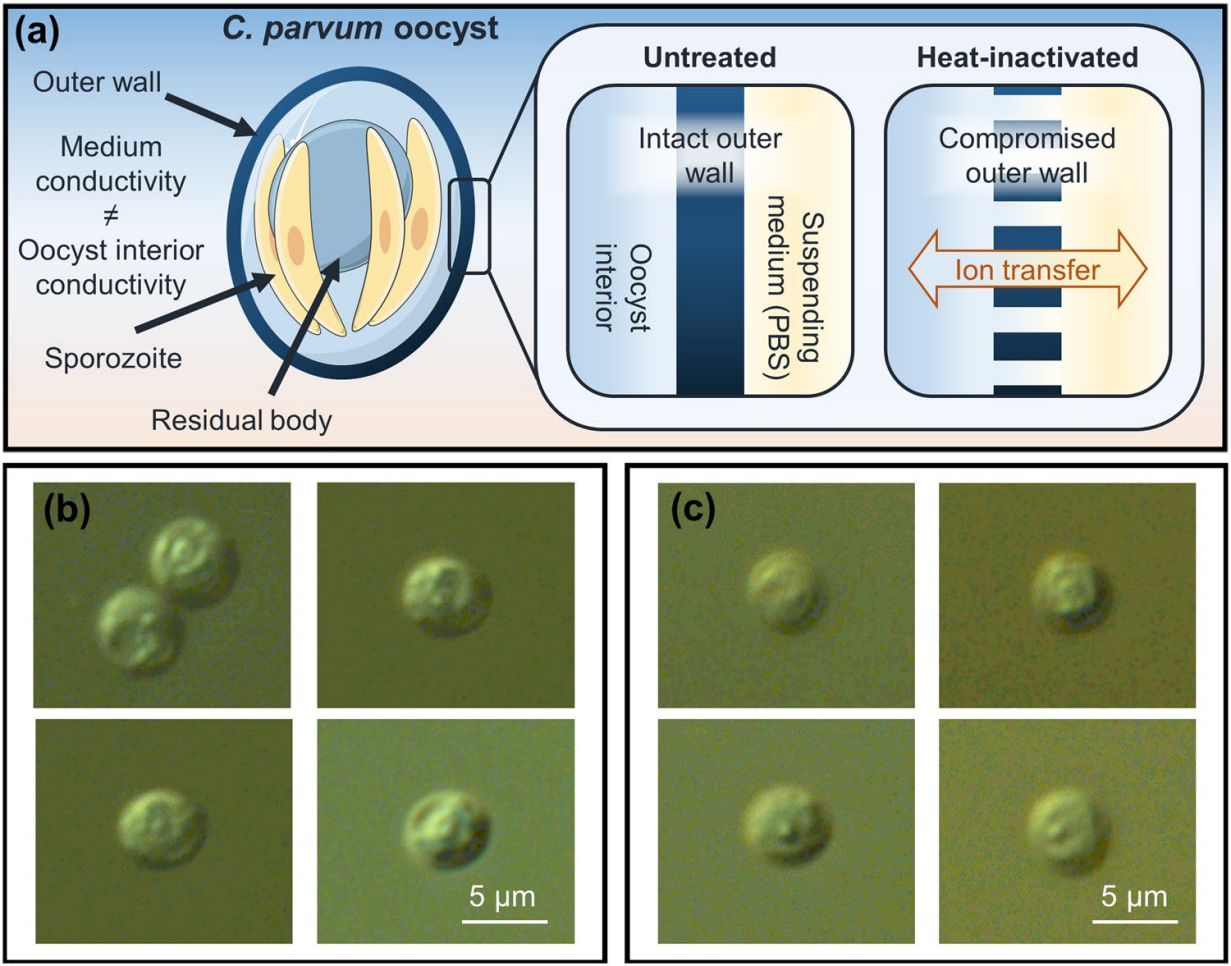
Diagram showing the structure of both the untreated (live) and heat-inactivated (non-viable) *C. parvum* oocysts. (**a**) Schematic showing hypothetical effect of heat-inactivation on oocysts suspended in PBS. The *C. parvum* oocyst generally consists of a trilaminar outer wall, which contains four naked, sporozoites and a membrane-enclosed residual body. Upon inactivation, the direction of ion transfer is influenced by buffer conductivity/osmolality. (**b**) Microscope images of untreated and (**c**) heat-inactivated oocysts (in PBS), viewed with oil immersion (100x) DIC microscopy.

The disruption of the selectively permeable membrane of a biological cell is typically measured using a viability stain such as Propidium Iodide (PI). Upon entering cells with compromised membranes, PI binds to DNA, intercalating between the bases which increases the fluorescence significantly. If a compromised cell membrane is permeable to PI, ions will be able to move freely across (*i.e*., the cell is electrically leaky, Fig. 3), which manifests as changes in the low frequency impedance^34^. Therefore, non-viable cells may appear smaller in electrical volume but not optical volume. Non-viable cells become incapable of regulating ion transfer and maintaining osmotic pressure as efficiently as viable cells^34, 41^, as, in the case of a *C. parvum* oocyst, is shown diagrammatically in Fig. 3a. A contributing factor in the ability of viable oocysts to persist in the environment for long durations is their strict regulation of ion transfer. Upon inactivation, oocysts no longer regulate ion transfer as efficiently as viable oocysts. Thus, the internal composition of the inactivated oocyst may become compromised. For example, the microscope images of untreated (Fig. 3b) and heat-inactivated (Fig. 3c) *C. parvum* show that the interior constituents appear less granular in heat-inactivated oocysts, which may indicate a change in their internal composition. Therefore, by suspending oocysts in non-isosmotic conditions, it may be possible to induce change in the ionic composition of an oocyst and consequently change its intermediate and/or high frequency impedance.

### Impedance Analysis of Parasites

**Figure 4:**
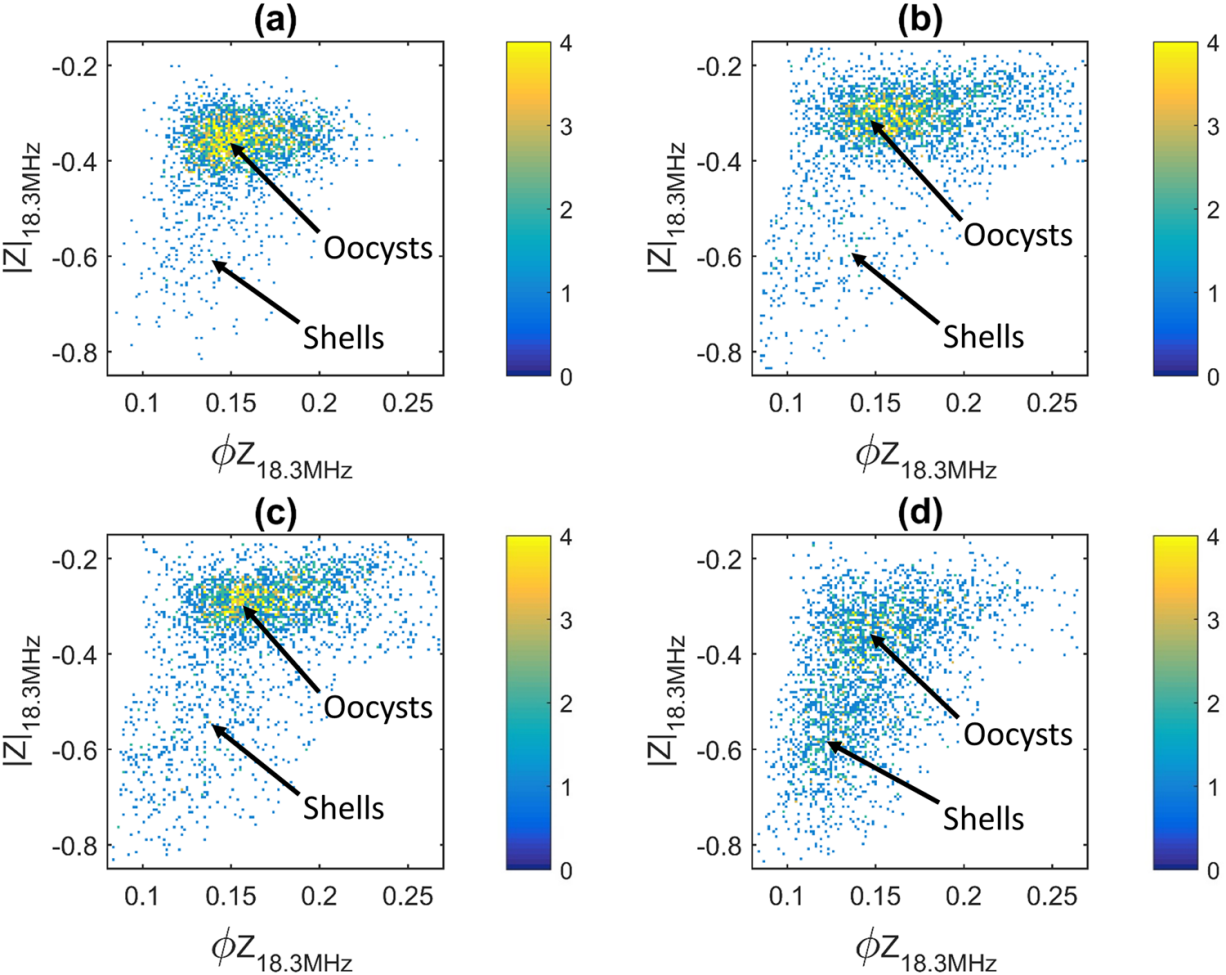
Example impedance scatter plots for four untreated *C. parvum* samples at a frequency of 18.3 MHz measured in 0.5x PBS of conductivity σ_m_ = 0.76 S m^−1^. Each data point is a single oocyst and colour represents data density. The first 3000 detected events are plotted in each figure subset and oocyst and shell populations are annotated as per the analysis described in the modelling section. (**a**) Calf-sourced oocysts (Iowa strain) measured within 1 month of oocyst propagation. (**b**) Mouse-propagated oocysts (Iowa strain) used within 1–2 months. **(c)** Different sample of calf-sourced oocysts (Iowa strain) used within 1–2 months. (**d**) Calf-sourced *C. parvum* (Moredun strain) measured within 2–3 months.

Figure 4 shows experimental impedance data for four different untreated *C. parvum* populations suspended in 0.5 × PBS (σ_m_ = 0.76 S m^−1^), at a reference frequency of 18.3 MHz. A high reference frequency was selected so that the internal properties of the oocysts could be probed^31, 34^. Impedance magnitude (|Z*|*) is plotted on the *x*-axis and impedance phase (ΦZ) on the *y*-axis, where each data point represents a single oocyst and its colour reflects the data density.

With environmental aging, *e.g*., exposure to natural stress like UV light and temperature variations or alternatively, sample transit and processing when used in a laboratory setting, some oocysts may become collapsed, distorted or even excysted^42^. In these cases, the infective sporozoites may be lost and consequently oocysts become “empty shells” or “ghosts”. Empty shells are routinely detected in environmental drinking-water samples^43^ and the impedance data in Fig. 4 shows the presence of such damaged/excysted oocysts. As is demonstrated in the next section, the shells appear as a separate cluster below the main oocyst population and have lower impedance magnitude (as they are electrically leaky) and phase on average. The subplots of Fig. 4a–d are in ascending order of age. Interestingly, the youngest (a) and oldest (d) *C. parvum* samples have the least and most shells present respectively (% shells in a = 6, b = 6, c = 8 and d = 41), which implies that the number of shells increases with environmental age.

### Impedance Modelling

Generally, the dielectric behaviour of biological cells in suspension is defined by Maxwell’s mixture theory (MMT)^44^, which describes the relation between the complex impedance of the suspension, the cell properties, the suspending medium and the volume fraction^45^. Based on MMT, shell-based models have been used to model the dielectric properties of cells^45–47^. Single shell models describe cells with just a membrane and no interior (see Fig. 2), and double shell models are applied for more complex cells with interior organelles. The frequency-dependent behaviour of these models can be plotted in terms of Debye relaxations^48^. Single shells have a single relaxation and double shells have two (the first associated with the cell membrane and the second with the interior organelles, Fig. 2b)^27, 45^. Thus, the integrity and complexity of a cell can be inferred by fitting data to these models.

**Figure 5:**
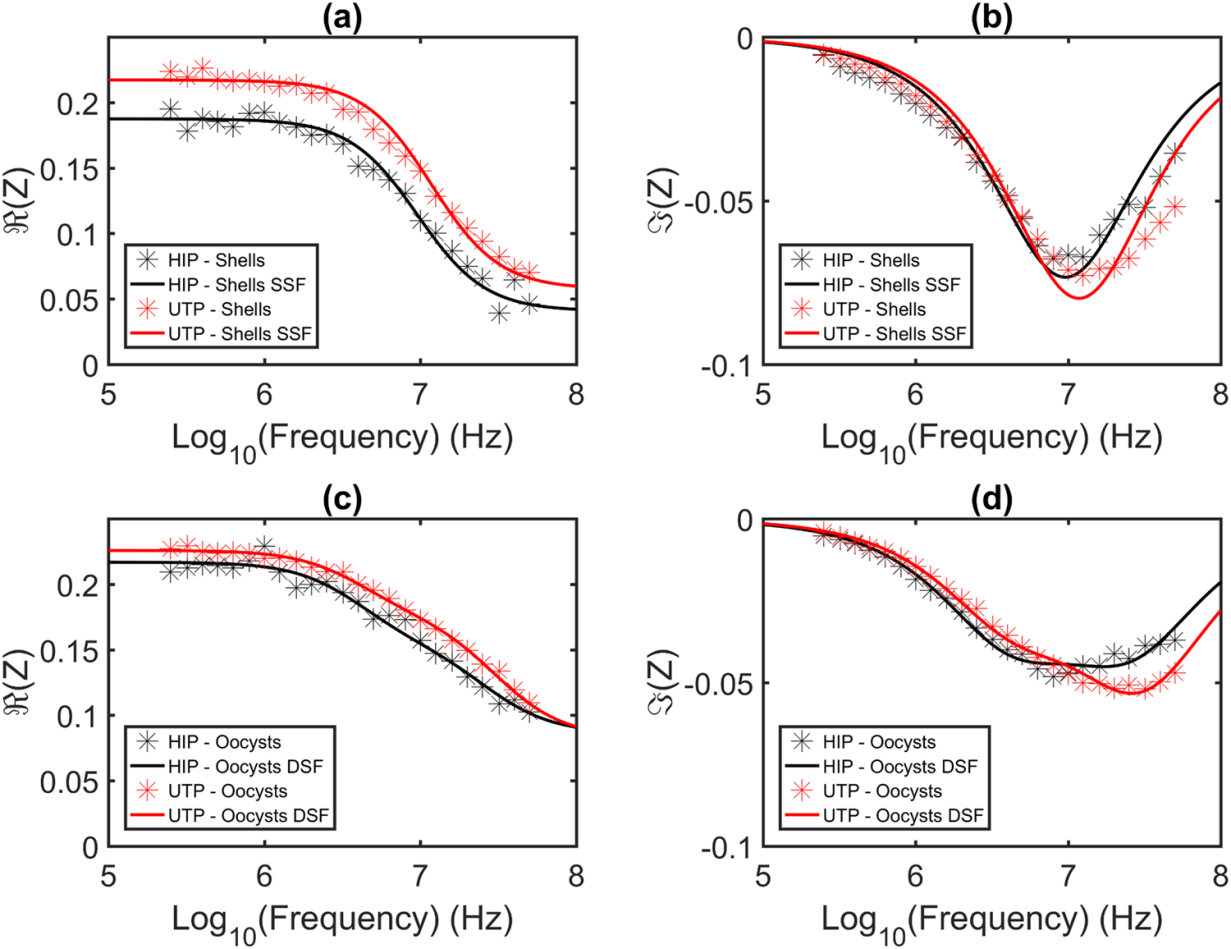
Impedance spectrum for untreated (UTP) and heat-inactivated *C. parvum* (HIP) in the frequency range 250 kHz to 50 MHz, for a buffer conductivity of σ_m_ = 0.76 S m^−1^. The mean value of the real ($\Re$(Z)) and imaginary ($\Im$(Z)) parts of impedance for shells ((**a**) and (**b**)) and oocysts ((**c**) and (**d**)), respectively, is plotted (stars). The two sets of data were then modelled using a single shell fit (SSF) for shells ((**a**) and (**b**)) and a double shell fit (DSF) for oocysts (**(c)** and **(d)**), and plotted over the data (solid lines).

The frequency dependent impedance for the average population of untreated and heat-inactivated *C. parvum* oocysts was measured from 250 kHz to 50 MHz, with a buffer conductivity of σ_m_ = 0.76 S m^−1^ - see Fig. 5. Each data point represents the mean of 1,000 oocysts per probe frequency.

The impedance data for the empty shells in untreated and heat-inactivated *C. parvum* suspensions was modelled by fitting the data to a single Debye relaxation representing a single shell fit (SSF) – Fig. 5a,b. As expected, the simple structure of the oocyst shell, where no sporozoites or other organelles are present, fits well to a single relaxation. Moreover, heat-inactivation of the suspension results in physical disruption of the membrane, leading to an apparent reduction in the magnitude of the low frequency impedance (Fig. 5a). The main oocyst population could not be fitted to a single shell model (see Supplementary Fig. S2) meaning that this model does not fully represent the intricacies of the oocyst structure. The double shell model however, provides a good fit as shown in Fig. 5c,d, *i.e*., it correctly represents the relaxation the oocyst membrane (~1–10 MHz) followed by the relaxation of the interior content (>10 MHz). Furthermore, as seen for the shells, heat-inactivation and consequent outer wall disruption causes a small drop in low frequency impedance (Fig. 5c). Thus, the simple model confirms that the two populations (Fig. 4) are correctly identified and that the *C. parvum* shells can be excluded from future analysis of the oocysts.

### Optimal Suspension Conductivity

**Figure 6:**
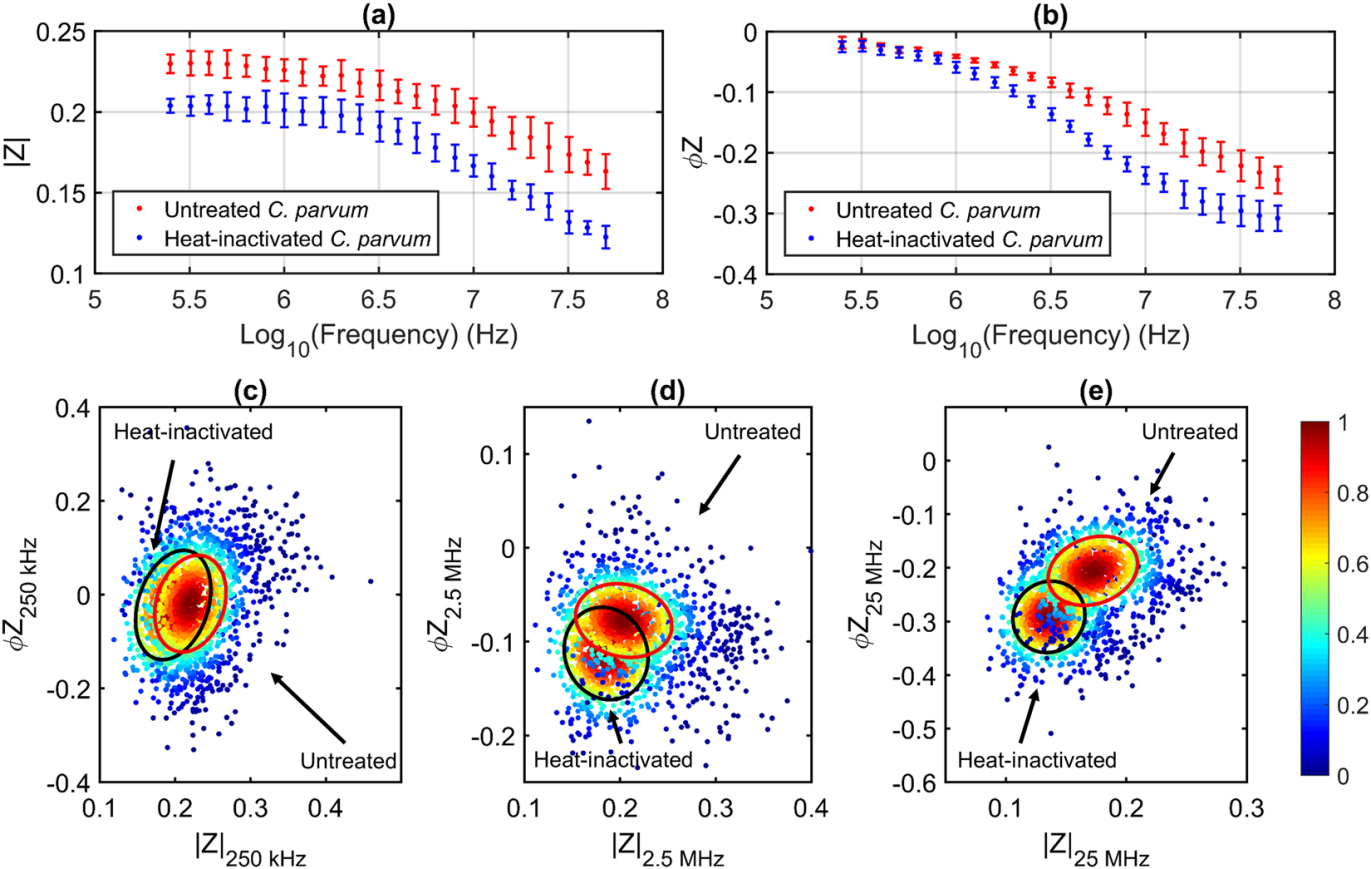
Impedance of *C. parvum* in PBS. (**a**) Impedance magnitude and (**b**) phase of untreated (red) and heat-inactivated (blue) *C. parvum* as a function of frequency; data is mean ± SD magnitude for all four *C. parvum* samples. The low, intermediate and high frequency impedance of untreated and heat-inactivated *C. parvum* are plotted together at (**c**) 250 kHz, (**d**) 2.5 MHz and **(e)** 25 MHz. The annotated confidence ellipses contain 50% of each population and the colour of each datapoint represents the normalized proximity of each event to the respective population mean. Untreated and heat-inactivated suspensions containing oocysts from all four stock samples were measured separately.

The average impedance of all untreated and heat-inactivated *C. parvum* populations suspended in a buffer of conductivity (σ_m_ = 1.51 S m^−1^) is shown in Fig. 6a,b. With increasing buffer conductivity, the relaxation frequency of the outer wall shifted towards higher frequencies as expected. Figure 6 also shows that the dielectric properties of untreated and heat-inactivated *C. parvum* are different, especially at higher frequencies (see Fig. 6c,e), where the signal is influenced by the properties of the oocyst interior. Generally, the *C. parvum* oocyst interior consists of four (nuclei-containing) sporozoites – the infective agents of the oocyst – and a membrane-enclosed residual body. The residual body contains a large lipid body, numerous amylopectin granules, a crystalline protein inclusion, ribosomes and cytomembranes^4^. For heat-inactivated *C. parvum*, the integrity and/or composition of the internal fluid and interior organelles was presumably compromised, and the osmolality difference between the oocyst interior and suspending medium was greater in 1xPBS than the 0.5x PBS buffer (σ_m_ = 0.76 S m^−1^) – see Fig. 5c,d. Thus, the difference in the high frequency impedance of the parasite populations was greater for those re-suspended in PBS (Fig. 6). These findings suggest that the parasites were exposed to hyperosmotic conditions when suspended in PBS. The data in Fig. 6a,b also indicates no difference in either the magnitude or phase, regardless of strain or source of the *C. parvum* oocysts.

**Figure 7:**
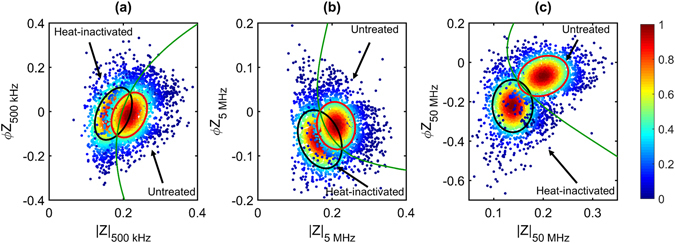
Impedance scatter plot for *C. parvum* in 5x PBS. Low, intermediate and high frequency impedance data for untreated and heat-inactivated *C. parvum* are plotted together at (**a**) 500 kHz, (**b**) 5 MHz and (**c**) 50 MHz. Annotated confidence ellipses contain ~50% of untreated or heat-inactivated populations (1,000 events plotted for each population). The green line indicates the equal probability boundary between the two populations. The colour of each data point represents the normalized proximity of the event to the respective population mean.

The suspension of oocyst populations in a high conductivity, hyperosmotic buffer (5x PBS; σ_m_ = 7.10 S m^−1^) gave rise to the largest difference in the impedance of untreated and heat-inactivated *C. parvum*. Figure 7 shows the low, intermediate and high frequency impedance of *C. parvum* in 5x PBS. High frequency impedance enables the clearest discrimination of the parasite populations and this difference is enhanced by increasing suspending medium conductivity. The green line indicates the line of equal probability deviation between the populations (*i.e*., where a detected event has equal chance of belonging to either population) and is an estimate of confidence in the discrimination between untreated and heat-inactivated *C. parvum*. For example, at 50 MHz, where the clearest discrimination was achieved (Fig. 7c), 91% of the events to the right of the equal probability boundary were from the untreated *C. parvum* population and 92% of the events to the left were from the heat-inactivated *C. parvum* population. Thus, MIC enabled the identification of untreated or heat-inactivated *C. parvum* with over 90% confidence in these experimental conditions. The data contained in Table S2 shows the identification confidence achieved for each individual sample and demonstrates that sample age and/or sample viability negatively affects the discrimination level.

### Species Analysis

The impedance of pathogens which are commonly isolated using EPA 1623.1 was measured to assess the suitability of MIC for species- and genus-level discrimination of waterborne parasites. Specifically, samples containing *C. parvum* (CP), *C. muris* (CM) and *G. lamblia* (GL) suspended in PBS (σ_m_ = 1.61 S m^−1^) were analysed. To properly gate and characterise the impedance of each population, samples were firstly measured independently – Supplementary Fig. S3. The cube root of the low frequency impedance magnitude is proportional to particle diameter^28, 34^. Using this data, the average diameter of the parasites was determined as CP = 4.3 ± 0.3 µm, CM = 5.9 ± 0.4 µm and GL = 9.3 ± 0.4 µm, which correlates well with dimensions obtained from optical imaging^49, 50^.

**Figure 8:**
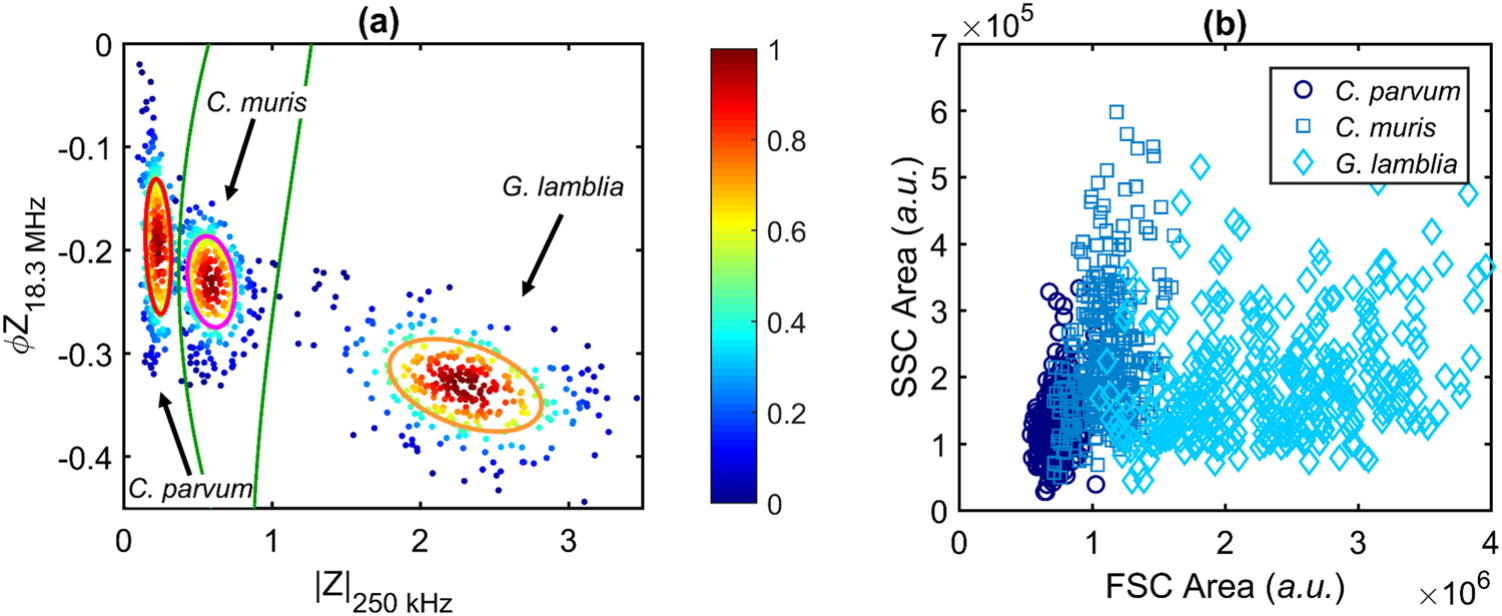
Impedance *vs* optical detection of parasites. (**a**) Scatter plot of phase at 18.3 MHz *vs* impedance magnitude at 250 kHz for all parasite species measured together in a mixed sample. Annotated confidence ellipses contain ~50% of each population. Green lines indicate boundaries defining equal probability deviation between adjacent populations. The colour of each data point represents the normalized proximity of the event to the respective population mean. (**b**) Conventional flow cytometry data (SSC *vs* FSC) for all parasite species in PBS. Individual optical scatter data for each population plotted together. A total of 1,000 events are plotted in both (**a**) and (**b**).

The difference in impedance between populations can be visualised in a number of ways, but the clearest discrimination is obtained from a scatter plot of the high frequency phase (at 18.3 MHz) against low frequency impedance magnitude (at 250 kHz), as shown in Fig. 8. This data effectively plots variations in internal structure/composition *vs* size. The figure shows data for 1,000 events which were obtained by measuring a sample that contained all the different populations. Using the same gates as in Figs 5 and 6, it was possible to identify CP with 98% confidence, CM with 93% confidence and GL with over 99% confidence. These results suggest that impedance analysis allows discrimination of these pathogens with a high degree of confidence, especially when compared to conventional flow cytometry (Fig. 8b).

The two species *C. parvum* and *C. hominis* are estimated to be responsible for over 90% of human cases of cryptosporidiosis^51^. However, other species (*C. meleagridis*, *C. canis, C. cuniculus* and *C. felis*) have also been associated with human infection and are considered “major human-pathogenic species”^49, 52^. In terms of oocyst dimensions, all these “major” species show similarity and are typically reported as elliptical oocysts within the size range of 4.0–5.0 × 5.0–5.5 µm^49^. Our results suggest that MIC may be able to discriminate oocysts of this size, with a high degree of certainty, from other species of *Cryptosporidium* that are not within this size range and which pose little or no risk to human health (as we have demonstrated with *C. parvum* and *C. muris*). Such species include (but not limited to) the larger *C. andersoni*, *C. baileyi* and *C. muris*, plus the smaller *C. xiaoi* and *C. ryanae* ^49^, which were all identified among the low-risk, contaminating species of UK drinking-water over a one-year period^53^. However, analysis of distinct species which have similar dimensions is required to assess whether inter-species variations in oocyst wall and interior structure/composition may facilitate impedance-based discrimination of such oocysts.

## Conclusion and Outlook

Utilisation of MIC offers for the first time the ability to assess the species and viability status at the single (oo)cyst level, with the additional advantages of rapid automated processing and a label-free, non-destructive method. The impedance properties of *C. parvum* oocysts of varying source, strain and age were measured in buffers of different conductivity. The impedance data of the oocyst and shell populations within each sample were fitted to either a single or double shell model. The results indicated that it was possible to identify the presence of damaged/excysted oocysts (termed shells) and that shell numbers increased with environmental age.

The ability of MIC to probe different layers of the oocyst structure means that impedance analysis enables clearer discrimination than optical flow cytometry – an equivalent, label-free method (see Supplementary Fig. S4). The data shows that untreated and heat-inactivated *C. parvum* can be identified with over 90% certainty at high frequencies when suspended in 5x PBS. It is hypothesised that the contrast is best in this conductivity because the non-viable parasite loses the ability to regulate ion transfer, resulting in an inward flux of ions which increases the electrical “contrast” between untreated and heat-inactivated *C. parvum*. In addition, single *C. parvum*, *C. muris* and *G. lamblia* (oo) cysts were identified with over 92% certainty from the high frequency phase *vs* low frequency magnitude data (when suspended in PBS).

This technology could potentially be useful for water utilities in, *e.g*., a warning system which more rapidly identifies single *Cryptosporidium* and *Giardia* (oo)cysts post-recovery *via* EPA 1623.1. As demonstrated, the system can also simultaneously provide an indication of the viability and species of recovered (oo)cysts, which may reduce the requirement for skilled microscopists and the associated labour demands. This system could also be used for foodborne protozoan monitoring or the analysis of veterinary or clinical samples.

For widespread use of this technique in, *e.g*., water monitoring, this study should be extended to demonstrate that *Cryptosporidium* oocysts which have been inactivated by means other than heat-inactivation can also be discriminated from viable oocysts. Future work should also assess the suitability of MIC for discriminating other species of *Cryptosporidium*, which pose/do not pose a significant risk to human health as well as characterise the performance with (oo)cysts from different matrices, including water, food and faecal samples. The method should also be compared for replacement of the “gold standard” animal infectivity protocols.

Subject to further validation with environmental samples, this technology could be implemented into the existing regulatory framework for waterborne protozoan monitoring to enable rapid assessment of oocyst viability and thus provide a more accurate assessment of the risk posed to human health. For example, the existing detection method utilises immuno-magnetic separation (IMS) for parasite enrichment, giving a ~50 µL volume with recovered oocysts^16^. By increasing the conductivity of this product sample, it may be possible to discern between untreated and heat-inactivated *C. parvum* using MIC. The system could operate at an increased flow rate^28^, therefore it would be possible to process a sample in much less than an hour. Finally, it is also possible to view results in real-time, which would allow water utilities to rapidly identify potentially hazardous drinking-water.

## Electronic supplementary material

Analysis of Parasitic Protozoa at the Single-cell Level using Microfluidic Impedance Cytometry - Supplementary Information
