# Supplementary material for: Analysis of Parasitic Protozoa at the Single-cell Level using Microfluidic Impedance Cytometry
Source: Sci Rep. 2017 Jun 1;7:2601. doi: 10.1038/s41598-017-02715-y (PMC5454013; doi:10.1038/s41598-017-02715-y)
Supplement: Supplementary file 1 — Analysis of Parasitic Protozoa at the Single-cell Level using Microfluidic Impedance Cytometry - Supplementary Information [file 41598_2017_2715_MOESM1_ESM.doc]

**Analysis of Parasitic Protozoa at the Single-cell Level using Microfluidic Impedance Cytometry^†^**

J.S. McGrath^1#^, C. Honrado^2#^, D. Spencer^2^, B. Horton^3^, H.L. Bridle^1^ and H. Morgan^2*^

^†^*Electronic Supplementary Information available*

^1^*Institute of Biological Chemistry, Biophysics and Bioengineering, School of Engineering and Physical Sciences, Heriot-Watt University, Edinburgh, UK, EH14 4AS*

^2^*Faculty of Physical Sciences and Engineering and Institute for Life Sciences, University of Southampton, Southampton, UK, SO17 1BJ*

^3^*Moredun Scientific, Pentlands Science Park, Bush Loan, Penicuik, Midlothian, EH26 0PZ*

*^#^J.S.M. and C.H. contributed equally to this work*

*^*^Corresponding author. Telephone: +442380593330. E-mail: hm@ecs.soton.ac.uk.*

**Supplementary Information**


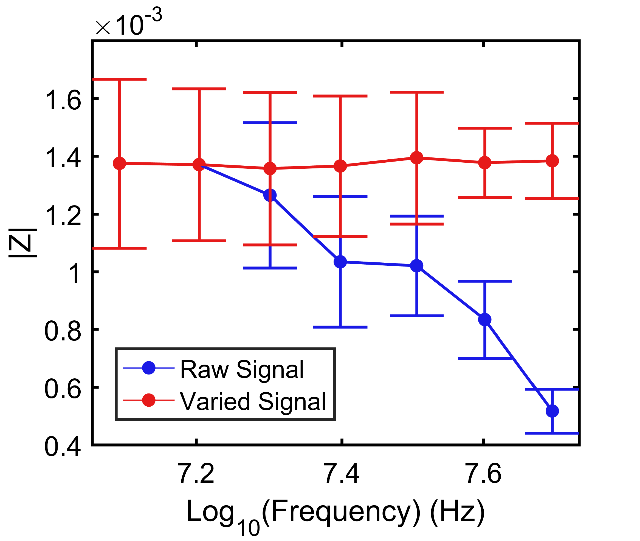


**Supplementary Figure S1:** Attenuation of signal loss. High frequency impedance magnitude of 7 µm polystyrene beads with (red) and without (blue) correcting for signal attenuation.

**Supplementary Table S1**. Excystation assay results for *C. parvum* samples.

| *Sample* | *Treatment* | *Viability (%)* | *Sp/ Sh* |
| --- | --- | --- | --- |
| WC1 | Untreated | 95 | 3.1 |
|  | Heat-inactivated | 9 | 0 |
| WM | Untreated | 87 | 2.7 |
|  | Heat-inactivated | 18 | 0 |
| WC2 | Untreated | 86 | 2.9 |
|  | Heat-inactivated | 12 | 0 |
| MC | Untreated | 88 | 2.2 |
|  | Heat-inactivated | 33 | 0 |

*Notes: See sample preparation section for details of sample abbreviations.*

*Sp = sporozoite, Sh = shell.*


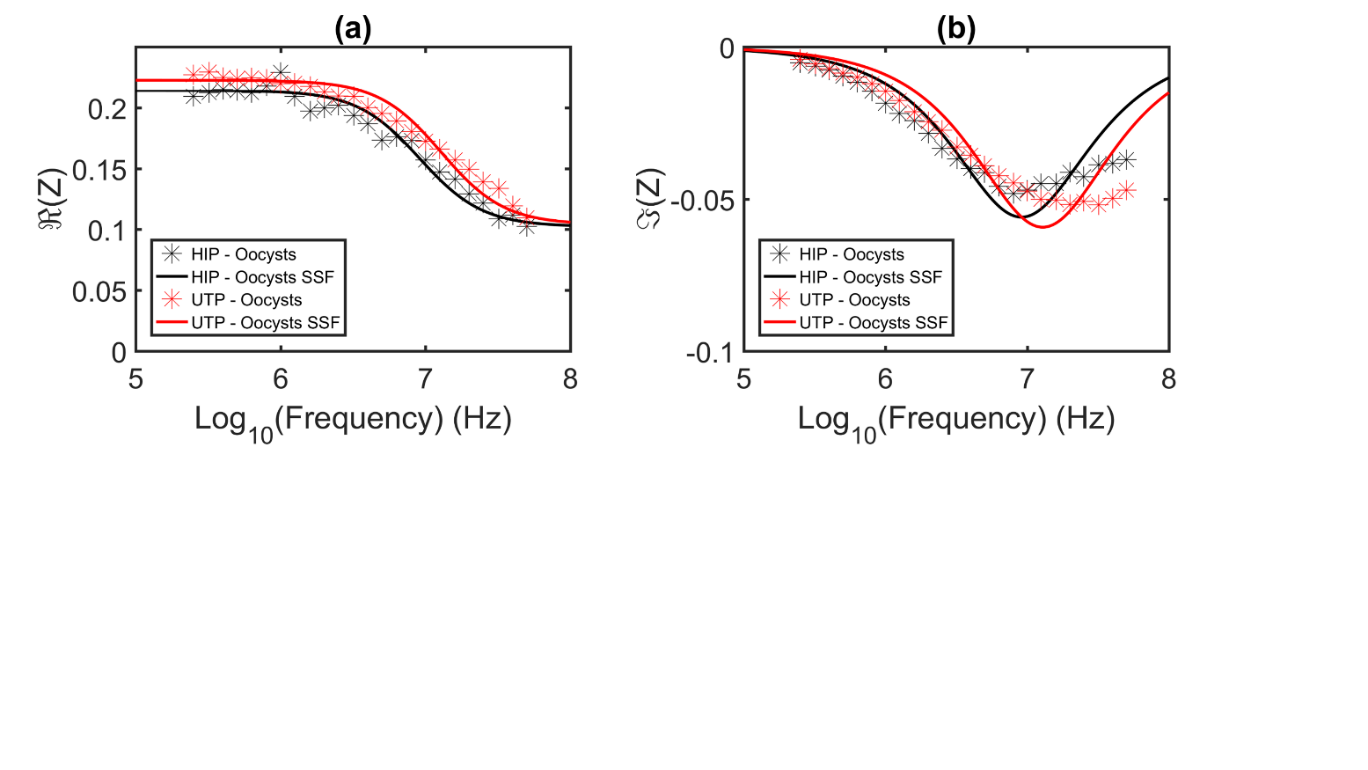


**Supplementary Figure S2:** Single shell fit modelled to UTP and HIP impedance data along the probe frequency range - 250 kHz to 50MHz, in a buffer conductivity of σ_m_ = 0.76 S m^-^¹. The mean values of the real (
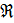
(Z)) and imaginary (
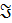
(Z)) parts of impedance for UTP **(a)** and HIP **(b)** are plotted (stars). The modelled single shell fits are indicated by solid lines.

| **Supplementary Table S2:** Identification confidence for untreated or heat-inactivated *C. parvum* oocysts from samples of different age using electrical impedance data obtained at a frequency of 50 MHz in 5x PBS. | | | | |
| --- | --- | --- | --- | --- |
| *Sample*  *(n = 250)* | *Sample age (months)* | | *Identification confidence (%)* | |
|  |  | *Untreated* | | *Heat-inactivated* |
| WC1 | 0-1 | | 95 | 97 |
| WM | 1-2 | | 85 | 84 |
| WC2 | 1-2 | | 87 | 86 |
| MC | 2-3 | | 88 | 90 |

*Notes: Abbreviations explained in sample preparation section; n is sample size.*

**
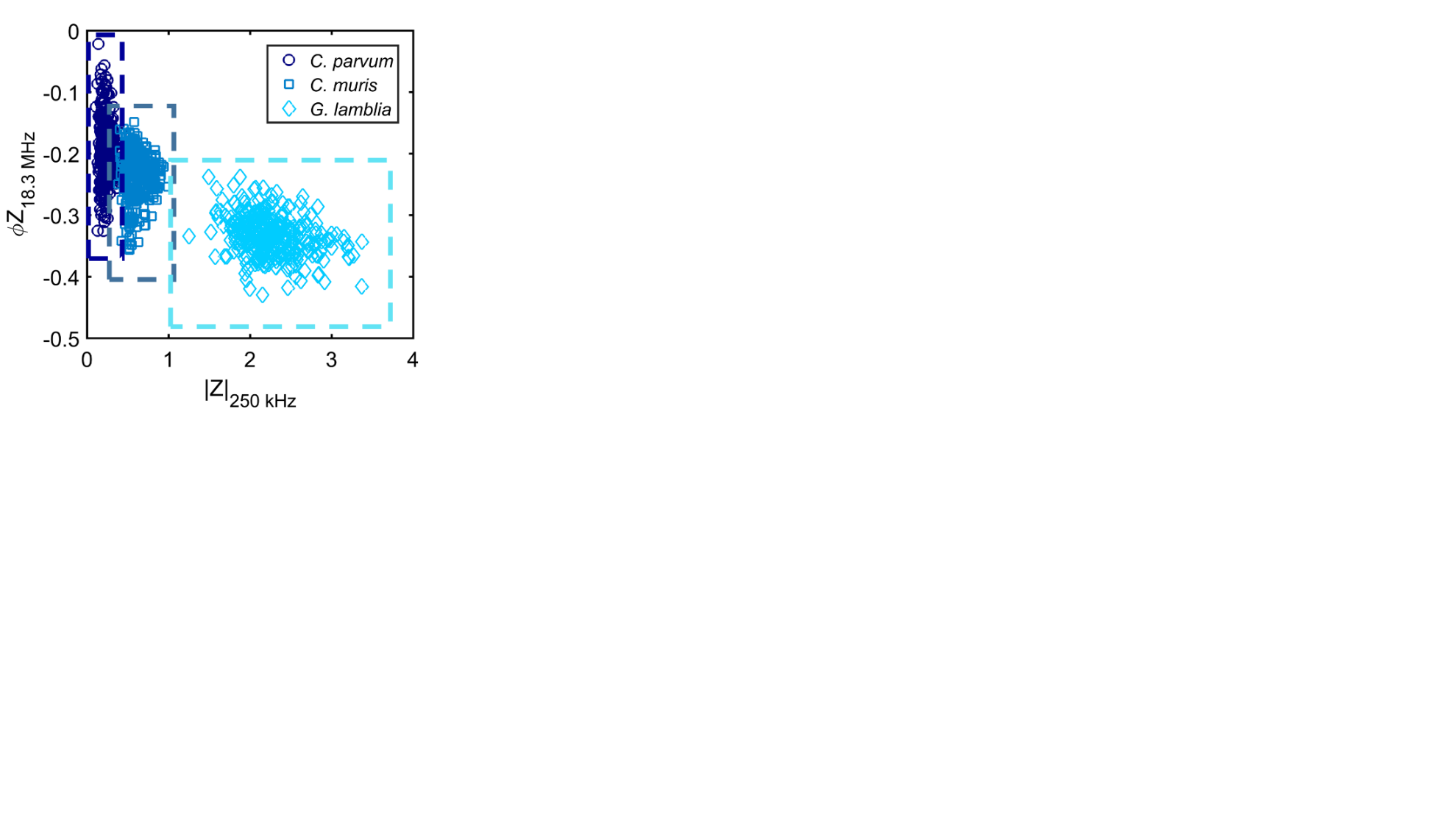
**

**Supplementary Figure S3:** Low frequency magnitude (250 kHz) *vs* high frequency phase data (18.3 MHz) for parasite suspensions. Impedance data for each population was measured independently but is plotted together – 1000 events are plotted. The gates (colour-coded, dashed lines) devised from independent sample analysis were used as a reference for future, mixed sample analysis.


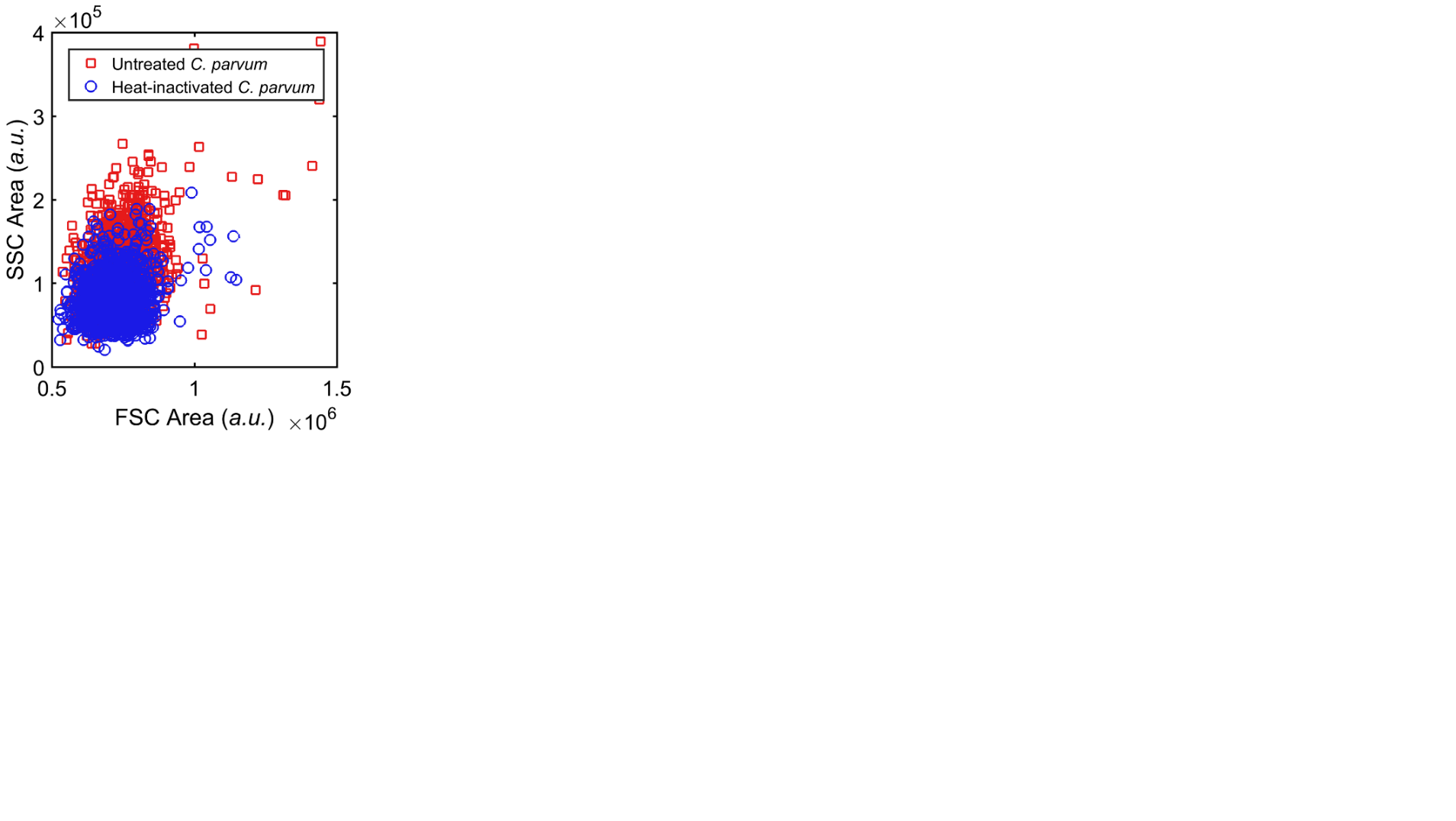


**Supplementary Figure S4:** Conventional flow cytometry data (SSC *vs* FSC) for untreated and heat-inactivated *C. parvum* in PBS. Individual optical scatter data for viable or non-viable *C. parvum* are plotted together.
